# *In situ* Responses of the Eelgrass *Zostera marina* L. to Water Depth and Light Availability in the Context of Increasing Coastal Water Turbidity: Implications for Conservation and Restoration

**DOI:** 10.3389/fpls.2020.582557

**Published:** 2020-12-15

**Authors:** Shaochun Xu, Pengmei Wang, Feng Wang, Peng Liu, Bingjian Liu, Xiaomei Zhang, Shidong Yue, Yu Zhang, Yi Zhou

**Affiliations:** ^1^CAS Key Laboratory of Marine Ecology and Environmental Sciences, Institute of Oceanology, Chinese Academy of Sciences, Qingdao, China; ^2^Laboratory for Marine Ecology and Environmental Science, Qingdao National Laboratory for Marine Science and Technology, Qingdao, China; ^3^Center for Ocean Mega-Science, Chinese Academy of Sciences, Qingdao, China; ^4^CAS Engineering Laboratory for Marine Ranching, Institute of Oceanology, Chinese Academy of Sciences, Qingdao, China; ^5^College of Earth Sciences, University of Chinese Academy of Sciences, Beijing, China

**Keywords:** seagrass meadow, depth limit, light requirement, response, methodology, reproduction, restoration, *Zostera marina*

## Abstract

Accelerating losses of seagrass meadows has led to efforts to restore these highly productive and beneficial ecosystems globally. Depth and light availability are critical determinants of seagrass restoration success. Eelgrass (*Zostera marina* L.) is the dominant seagrass species in the temperate northern hemisphere, but its global distribution has reduced dramatically. The main aims of this study were to determine: (1) the depth limit for *Z. marina* survival in Ailian Bay, north China, and (2) how light availability affects the growth and recruitment of *Z. marina* as a basis for identifying a suitable depth range for successful restoration. To achieve these aims, *Z. marina* shoots were transplanted from a nearby donor site, Swan Lake, to an experimental site, Ailian Bay, and the temporal responses of *Z. marina* shoots to light availability at water depths ranging from 1 to 8 m were investigated using *in situ* suspended cultures. Four suspended shoot transplantation experiments were conducted in 4 years. The results showed that the transplanted *Z. marina* shoots could survive and branch during an annual growth cycle, permanently underwater, at a depth ≤3 m. Due to the local turbidity of the waters in Ailian Bay, a depth of 4 m led to sufficient light deprivation (reduced to 6.48–10.08% of surface irradiance) to negatively affect seagrass shoot density and clonal reproduction. In addition, reproductive shoot density also tended to decline with water depth and light deprivation. Our results indicated that *Z. marina* population recruitment, through sexual and asexual (clonal growth) reproduction, were negatively affected by increasing water depth and light deprivation. These findings may provide a suitable depth range for the successful restoration of *Z. marina* in local coastal waters. They may also be applied to the management and restoration of *Z. marina* globally.

## Introduction

Seagrass meadows are among the most productive plant communities, providing habitats, food, and nurseries for a variety of marine organisms (Costanza et al., 1997; Verweij et al., 2008; Barbier et al., 2011; Liu et al., 2013; Unsworth et al., 2018b), regulating nutrients (Barbier et al., 2011), and functioning as key sites for global carbon storage in the biosphere (Fourqurean et al., 2012). Unfortunately, due to the impact of multiple stressors (environmental, biological, and climatological) (Collier and Waycott, 2009; Unsworth et al., 2018a), seagrass meadows have been declining since 1990 at a rate of 7% per annum (Waycott et al., 2009). With the development of coastal construction, the turbidity of coastal waters has been increasing, thus the light availability has been reducing (Elsahragty and Kim, 2015). Reduced light availability due to increased anthropogenic nutrient loading and sedimentation has been identified as one of the primary causes of seagrass loss (Jennifer et al., 2003; Orth et al., 2006). Furthermore, light is often considered one of the most critical factors that controls the distribution and growth of eelgrass (Lee et al., 2007; Zhang et al., 2020).

The eelgrass *Zostera marina* L. is the dominant seagrass species throughout the Atlantic and Pacific coasts of the temperate northern hemisphere (Green and Short, 2003). In China, eelgrasses are distributed in the coastal waters of Shandong, Hebei, and Liaoning provinces (Zheng et al., 2013; Xu et al., 2020a,b). Historical distribution information (1950–2013) of eelgrass specimens (Biological Museum, Chinese Academy of Sciences, Qingdao, China) and previous literature (Yang and Wu, 1984; Editorial Board of China Bay Survey, 1991; Ye and Zhao, 2002; Guo et al., 2010; Zheng et al., 2013) have revealed that a large number of eelgrass meadows having contracted sharply or disappeared (more than 80%). This is due to increased anthropogenic nutrient loading and sedimentation, according to the National Seagrass Resource Survey (2015–2020). Similarly, previously abundant *Z. marina* in Ailian Bay, north China, has declined dramatically and is currently mainly distributed in the sea cucumber ponds, according to local fishermen.

Faced with an increasing rate of eelgrass decline, eelgrass restoration throughout its distribution area in temperate northern hemisphere has become an important management tool to mitigate seagrass losses and to enhance critical ecosystem functions (Marion and Orth, 2010; Zhou et al., 2014; van Katwijk et al., 2016; Liu et al., 2019). Unfortunately, only 37% of seagrass restoration efforts have been successful in recent years (van Katwijk et al., 2016). Insufficient light availability has been identified as the one of the primary causes of eelgrass transplantation failure (e.g., Moore et al., 1997; van Katwijk et al., 2016); therefore, understanding their ability to acclimate to a range of light availability may be the key to ensuring their survival in restoration efforts (Eriander, 2017; Zhang et al., 2020).

The light requirements of seagrass have been investigated in many studies, and comprehensively reviewed by Lee et al. (2007); it has been revealed that the minimum light requirements of seagrass vary with species (Lee et al., 2007; Statton et al., 2018) and also within species depending on site-specific conditions (Lee et al., 2007). Therefore, it is important to establish the local minimum light requirement for individual species in an area of interest (Bulmer et al., 2016). The maximum depth at which seagrass grows provides an insight into the local light environment and the minimum light requirements of that seagrass. This parameter is also described as the maximum depth limit or depth of colonization. Seagrass coverage declines with water depth, and a light availability gradient exists along this natural depth distribution (Duarte, 1995). Depth has been shown to be a critical determinant of seagrass restoration success, suggesting it is imperative for identifying the most resilient areas that are most suitable for conservation and restoration (Aoki et al., 2020). Therefore, the depth of colonization of eelgrass in Ailian Bay needs to be confirmed for future restoration efforts.

To date, many studies have been performed to determine the light requirements of seagrass (Abe et al., 2003; Beck et al., 2018). Previous seagrass light requirements were estimated in field investigations (Abal et al., 1994; Abe et al., 2003; Ochieng et al., 2010; Zhang et al., 2020) and laboratory experiments (Abal et al., 1994; Moore and Wetzel, 2000; Eriander, 2017). Laboratory experimental systems remove important contextual factors of natural ecosystems (Crain et al., 2008), such as changes in water levels (due to tides and waves), episodic turbidity, and epiphyte growth which all contribute to the reduction of light availability to seagrass (Abal et al., 1994). Field investigations have been conducted in natural seagrass beds, but not necessarily in severely degraded areas. Moreover, responses of seagrass to light reduction are also dependent on site-specific conditions (Lee et al., 2007).

In the present study, an *in situ* suspended culture experiment was conducted in an area of interest, to directly examine long-term responses of transplanted *Z. marina* shoots to a depth gradient. Four suspended shoot transplantation experiments were conducted in 4 years. The aims of this study were to determine: (1) the depth limit for the survival of eelgrass (*Z. marina*) in Ailian Bay, and (2) how light availability affects the growth and recruitment of *Z. marina* as a basis for identifying a suitable depth range for successful restoration. The results from this study could provide important implications for future management and restoration of eelgrass, to avert further loss and enhance the potential for recovery. Additionally, *in situ* suspended seagrass cultures are described that may serve as a useful methodology for future field simulation experimentation.

## Materials and Methods

### Study Sites

#### Donor Site

**FIGURE 1:**
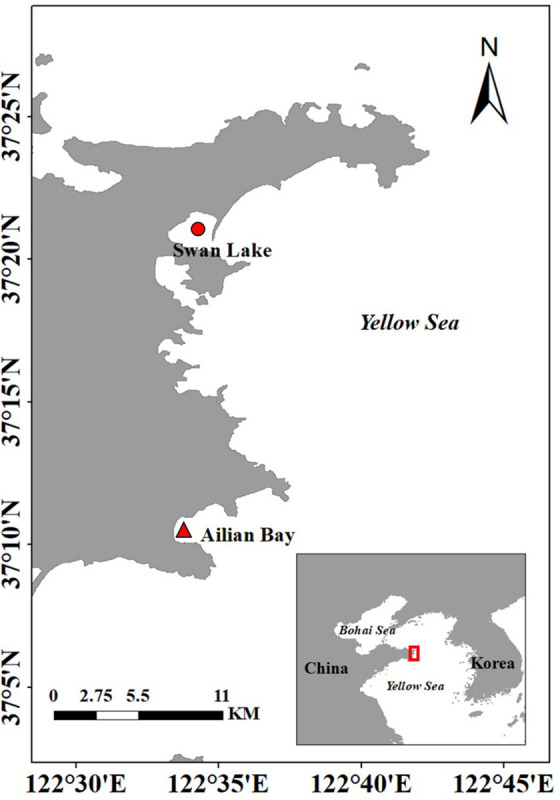
Location of the donor site in Swan Lake (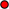) and the experimental site in Ailian Bay (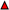), north China.

The donor site was located in Swan Lake, Rongcheng City, north China (Figure 1). Swan Lake is a 4.8-km^2^ marine lagoon that is connected to Rongcheng Bay by a narrow mouth, 86 m in width. The lagoon, a national nature reserve for the whooper swan, *Cygnus cygnus*, provides food resources and the largest wintering habitat for this bird in Asia (Wang et al., 2017). *Zostera marina* is distributed in the intertidal to subtidal zone, forming a meadow of ∼2.3 km^2^ (Zhou et al., 2015; Xu et al., 2019). The environmental conditions were shown in Table 1.

**TABLE 1:** Environmental conditions of the donor and experimental sites.

**Sites and locations**		**Environmental conditions**
Donor site	Swan Lake 122°34′E, 37°21′N	A marine lagoon. Salinity: 29.8–31.3 psu; annual average water temperature: 14.7°C; average depth: ∼2 m; irregular semidiurnal mixed tides (tidal range of ∼0.9 m) (Liu, 2012). Annual concentrations of NH_4_^+^, NO_3_^–^, NO_2_^–^, and PO_4_^3–^ were 2.14 ± 1.29, 1.31 ± 1.53, 0.15 ± 0.10, and 0.28 ± 0.17 μmol L^–1^, respectively (Zhou et al., 2015).
Experimental site	Ailian Bay 122°34′N, 37°10′E	A natural semi-enclosed bay. Salinity: 26.3–31.1 psu; annual average water temperature: ∼15.1°C; water depth: <14 m; irregular semidiurnal mixed tides (tidal range of ∼1.8 m). Annual concentrations of NH_4_^+^, NO_3_^–^, NO_2_^–^, and PO_4_^3–^ were 2.63 ± 0.88, 2.11 ± 1.34, 0.27 ± 0.07, and 0.31 ± 0.26 μmol L^–1^, respectively (Liu, 2012).

#### Experimental Site

The experimental site was located in Ailian Bay, Rongcheng City, ∼20 km from Swan Lake (Figure 1). It is a natural semi-enclosed bay. The bay is an important aquaculture area for north China (Yang et al., 2018), with more than 60% of it used for floating raft cultures of kelp or shellfish (scallops, oysters, and abalone). *Z. marina* is mainly distributed in sea cucumber ponds of this bay. The environmental conditions were shown in Table 1.

### Collection of Adult Plants

Adult plants were collected from the intertidal area in Swan Lake at low tide. *Z. marina* materials were collected carefully with shovels, and adult shoots that had at least 1–2 cm of rhizome with roots were selected. Twenty centimeters of leaf blade and leaf sheath were retained, and the extra part was removed by scissors (Zhou et al., 2014; Liu et al., 2019). The transplantation period ran from April to September (Table 2).

**TABLE 2:** Four suspended *Z. marina* shoot transplantation (from Swan Lake to Ailian Bay) experiments.

**Transplantation time**	**Experiment period**	**Depth gradient**	**Number of duplicates per depth**	**Initial shoot number per device**	**Monitoring time or frequency**	**Monitoring index**	**Experimental device**
Jun 2010	Jun 2010 to Jul 2011	2, 4, 6, and 8 m	4	12	Dec 2010 and Jul 2011	Shoot number and height	PE box (30 cm × 20 cm × 15 cm)
Apr 2011	Apr to Sep 2011	2, 4, 5, 6, and 7 m	4	12	Jul 2011	Shoot number and height	PE box (30 cm × 20 cm × 15 cm)
Sep 2011	Sep 2011 to Nov 2012	2, 3, 4, 5, and 6 m	4	20	Sep 2011 to Nov 2012; monitoring every 1–2 months	Shoot number and height	PE box (30 cm × 20 cm × 15 cm)
Apr 2014	Apr to Oct 2014	1, 2, 3, 4, and 6 m	5	10	Apr to Oct 2014; monitoring approximately monthly	Total and flowering shoot number, and shoot height	PVC pot (D = 20 cm, H = 12 cm)
							

### Transplantations

**FIGURE 2:**
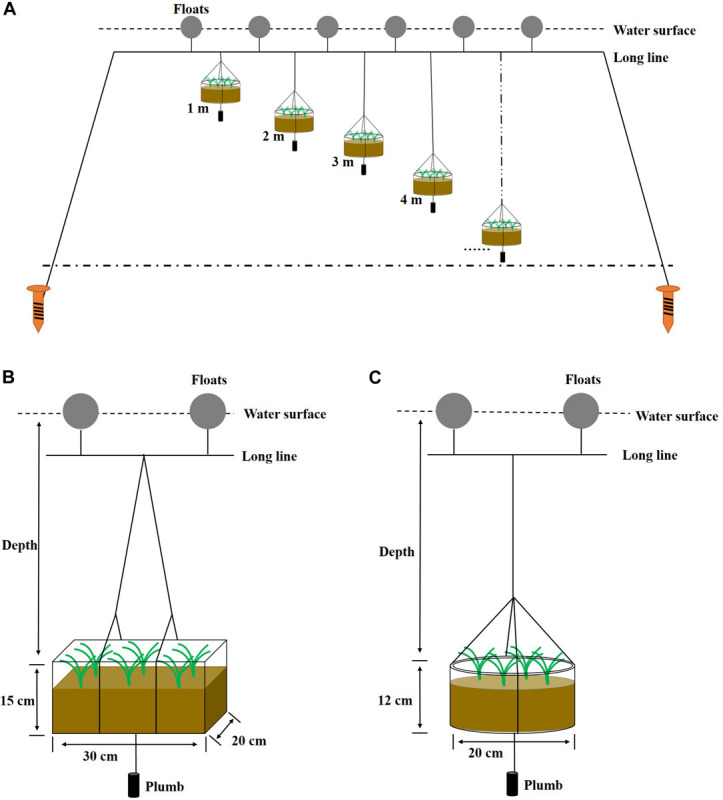
Floating raft **(A)**, PE box **(B)**, and PVC pot **(C)** used for suspended *Z. marina* transplantation experiments. PE boxes were used in 2010–2012, and PVC pots in 2014.

Four suspended shoot transplantation experiments were performed in 4 years (2010, 2011, 2012, and 2014; see Table 2). Shoots were transplanted using a stone anchoring method (Zhou et al., 2014). This method involves anchoring a transplanting unit (PU) consisting of three to four shoots with rhizomes and roots to a small elongate stone 50–150 g in weight using biodegradable thread or thin rope (e.g., cotton thread or hemp string). The small stones were collected from the seashore in Swan Lake. PUs were buried in an experimental device (PE box or PVC pot; Table 2 and Figures 2B,C), which contained a bottom layer of sediment taken from the bank of Swan Lake. The rhizomes were placed at a depth of 2–4 cm in sediments and on the side of the stone. The initial shoot number per device for the four experiments ranged from 10 to 20 shoots (Table 2). The experimental devices planted with *Z. marina* materials were then transported to Ailian Bay and tied to the rafts with polyethylene ropes (Figure 2A). Four transplantation experiments were conducted along a depth gradient (Table 2). To balance the experimental devices in the water, a one 1-kg plumb ball was tied to the bottom of each device.

### Monitoring Method

The main monitored parameters included shoot number and height in all four experiments, and flowering shoot number and shoot height were also monitored in 2014. Monitoring time and frequency were shown in Table 2.

### Environmental Parameters

Water temperature (°C), salinity (ppt), dissolved oxygen content (DO, mg L^–1^), pH, chlorophyll content (μg L^–1^), and depth (m) were measured using a multi-parameter water quality sonde (YSI 6600, United States). The parameters were measured over a 2–15 min period at each depth. The measuring sonde was moved up and down the water column while several measurements were recorded at various depths (1, 2, 3, 4, and 6 m), all within an hour. Variations in water parameters with depth were measured five times in total during April, May, June, August, and November 2014.

Light availability (μmol photons m^–2^ s^–1^) was measured using an ECO-PARSB sensor (Sea-Bird Scientific, United States). The light sensor was moved up and down the water column with light availability measurements recorded at various depths (0, 1, 2, 3, 4, and 6 m), all within an hour. Light intensity was obtained at four different days/times in total, in May, August, and October 2014, and the light intensity was measured every minute.

### Data Analyses

For the shoot transplantation experiment beginning in April 2014, differences among seagrass variables at the various depths were tested using a one-way ANOVA, and the specific treatment differences were identified using independent *t*-test. Simple linear regression was used to test the significance of the relationships between water depth and temperature. Before the analyses, the homogeneity of variance was tested using Levene’s test. Differences were considered significant at a probability level of *p* < 0.05. SPSS 20.0 was used for all data analyses. Simple linear regression analyses were considered significant at a probability level of *p* < 0.05.

#### Seagrass Responses to Transplantation Depth

To quantitatively analyze the responses of viable transplanted shoots to water depth and time from deployment in the 2014 experiment, the following linear model (see Zhang et al., 2020) was applied:

(1) S⁢(z)=a⁢z+b

Where S(z) is shoot density, z is depth at each time point of each year, and a and b represent equation coefficients. Here, S(z) is the vegetative shoot density, not the total shoot density, because vegetative shoot density is more suitable for the evaluation of lateral shoot branching, and reproductive shoots disappear in August every year. Any depths at which the measured shoot density overlapped with zero were excluded from linear model fitting. All linear regressions were performed using R version 3.6.3.

Such analyses were not performed for reproductive density or shoot height because quantitative relationships, such as those expressed in equation (1), could not be identified from a visual inspection of the data.

#### Light Variation With Depth

To explore whether water clarity varied spatially, and thus determine if the depth limits obtained from the analysis were affected by any spatial variations in water clarity, the spatial correlation of water turbidity with depth was assessed (see Zhang et al., 2020). This was achieved using non-linear regression to fit each of the four distributions for the dependence of light intensity on depth to Beer’s Law (Kirk, 1985):

(2) I=I⁢e0⁢x⁢p⁢(-K⁢zd*)

where, *I* represents the light availability at water depth *z* (m), *I*_0_ represents the surface light, and *K_*d*_^∗^* represents the light attenuation coefficient (m^–1^), which was not corrected for the sun’s location in the sky.

#### Improvement of the Experimental Device

Some PE boxes were overturned by marine currents during 2010–2011; therefore the design parameters of the experimental device were altered. In order to reduce the impact of currents on the experimental device, improvements to the stability of the device in seawater were attempted by reducing the drag force. The drag force is related to fluid density, frontal area of object, drag coefficient, and the velocity of fluid, and the shape of an object has a large effect on the amount of drag (Batchelor, 1967). This can be expressed as follows:

(3) $$F_{d}=0.5\,\rho \,\mu^{2}\,C_{d}\,A$$

where, *F*_*d*_ is the drag force, ρ is the mass density of the fluid, μ is the flow speed of the object relative to the fluid, *C*_*d*_ is the drag coefficient, and *A* is the frontal area.

In order to change the shape (reducing drag coefficient) and reduce the frontal area of the experimental device, PVC pots were alternatively used in 2014. The drag coefficient ranges of the PE box (cuboid) and PVC pot (short cylinder) were 1.05–2.05 (Aaronaes et al., 2015) and <0.64 (Mohammed et al., 2016), respectively.

## Results

### Suspended Shoot Transplantation Experiment

**FIGURE 3:**
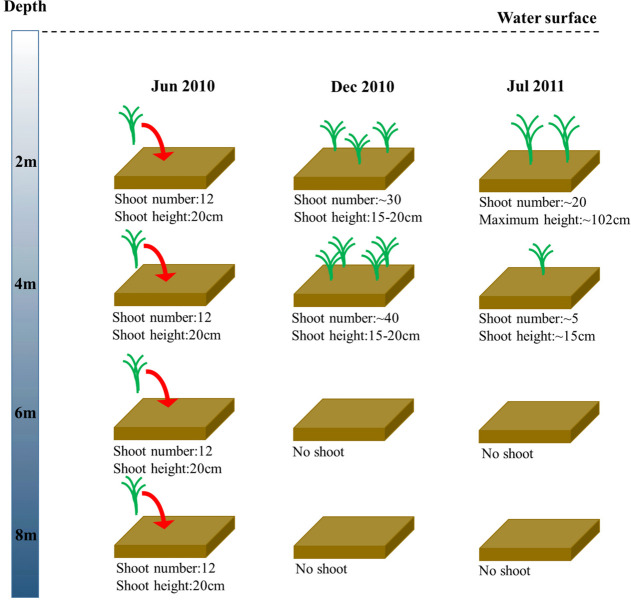
Changes in *Z. marina* shoot number and shoot height along a depth gradient in Ailian Bay in a shoot transplantation experiment beginning in June 2010.

In the shoot transplantation experiment beginning in June 2010, shoot densities at 2 m and 4 m increased by more than 100%, while there was no *Z. marina* remaining at 6 m and 8 m after approximately half a year of experimentation (Figure 3). After approximately a year of experimentation, the transplanted *Z. marina* at 2 m and 4 m successfully completed a 1-year life cycle, and reproductive shoots, with a maximum length of 102 cm, were found at 2 m. The shoot density at 2 m was four-fold greater than that at 4 m. There was also no *Z. marina* remaining at 6 m and 8 m after approximately a year of experimentation.

**FIGURE 4:**
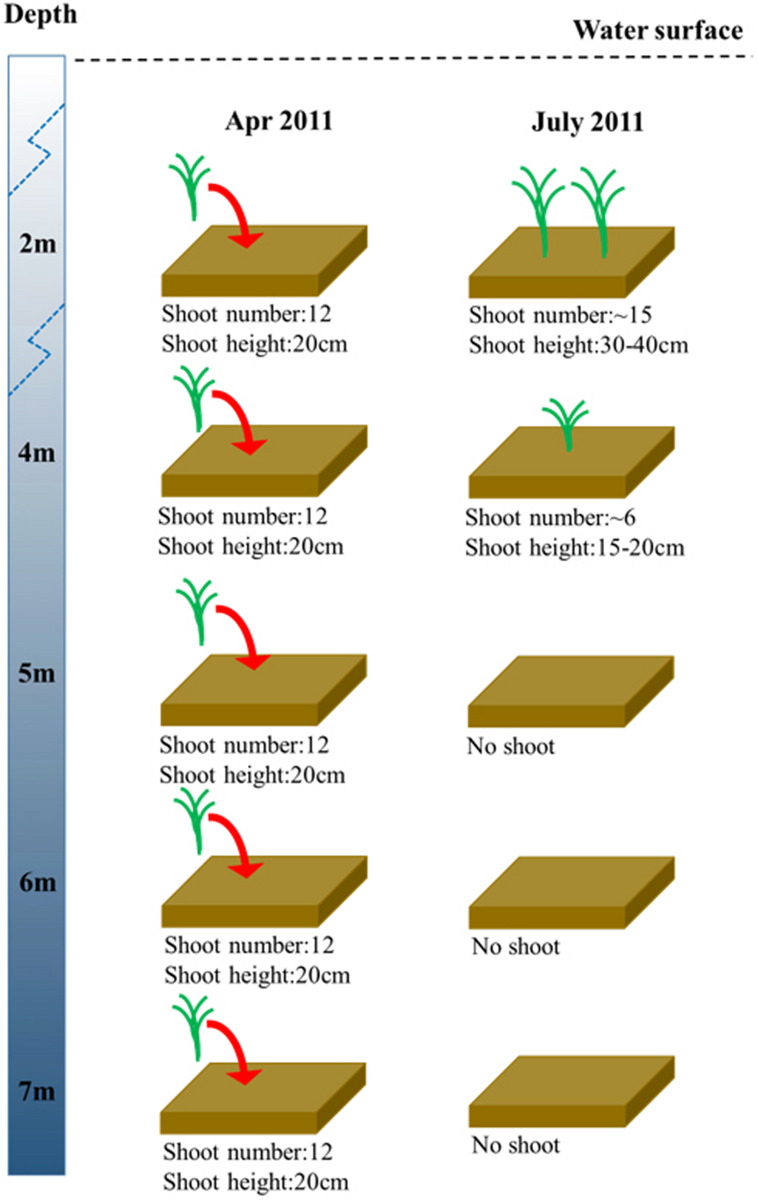
Changes in *Z. marina* shoot density and shoot height along a depth gradient in Ailian Bay in a shoot transplantation experiment beginning in April 2011.

In the shoot transplantation experiment beginning in April 2011, in order to test whether the depth between 4 and 6 m was suitable for eelgrass survival, *Z. marina* was transplanted at 5 m. After 3 months of experimentation, *Z. marina* survived only at 2 m and 4 m, and there was no *Z. marina* found at 5 m, 6 m, or 7 m (Figure 4).

**FIGURE 5:**
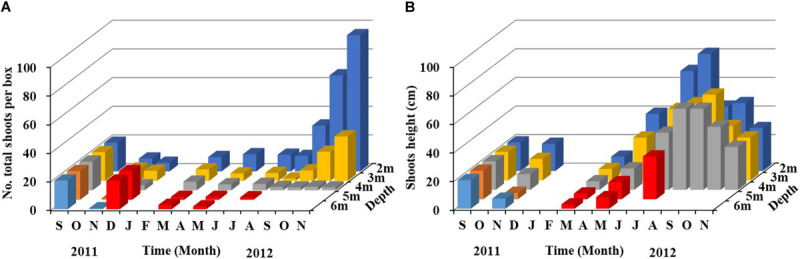
Temporal changes in *Z. marina* shoot density **(A)** and shoot height **(B)** along a depth gradient in Ailian Bay in a shoot transplantation experiment beginning in September 2011. Since *Z. marina* was not found at 5 m and 6 m in December 2011, 20 shoots per box were re-transplanted at 5 m and 6 m (red column). The values in the figure represent the means.

In the shoot transplantation experiment beginning in September 2011, shoot densities decreased gradually in autumn and winter, increased slightly in March 2012, and then maintained at a low level (Figure 5A). From September to November 2012, shoot densities at 2 m and 3 m increased rapidly, and shoot densities at 2 m and 3 m increased approximately three- to four-fold, indicating that lateral shoots were germinating and growing rapidly during this period. In November, there was ∼90 shoots per box at 2 m and ∼30 shoots per box at 3 m. Shoot density at 4 m maintained at a low level, with approximately two shoots per box in November, which was significantly lower than that at 2 m and 3 m. In December 2011, there was no *Z. marina* remaining at 5 m and 6 m; therefore, 20 shoots per box were re-transplanted at 5 m and 6 m; however, *Z. marina* had completely disappeared again in August.

Shoot height at 2 m, 3 m, and 4 m decreased in autumn and winter, and increased significantly from spring to summer (Figure 5B). Shoot height decreased with increasing water depth. The maximum shoot height at 2 m was ∼80 cm in August 2012, and ∼60 cm at 3 m and 4 m in September. Plant height began to decrease after summer, and it was ∼30 cm at the above mentioned depths in November.

**FIGURE 6:**
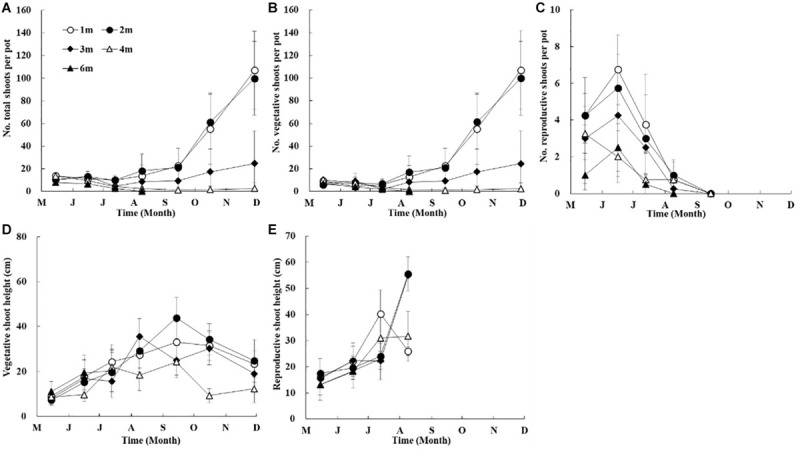
Temporal changes in *Z. marina* total shoot density **(A)**, vegetative shoot density **(B)**, reproductive shoot density **(C)**, vegetative shoot height **(D)**, and reproductive shoot height **(E)** along a depth gradient in Ailian Bay in a shoot transplantation experiment beginning in April 2014.

In the shoot transplantation experiment beginning in April 2014, total shoot densities at different depths were relatively stable in the first 3 months (Figure 6A). After the initial stable period, total shoot densities in shallow water (depths of 1 m and 2 m) increased rapidly, those at 3 m increased gradually, while those in deeper waters (depth >3 m) declined. The maximum total shoot densities at 1 m and 2 m were significantly higher than those at 3 m (*p* < 0.05), and approximately three-fold greater than those at 3 m. Very few shoots at 4 m depth survived until the end of the experiment, and shoots at 6 m depth had completely disappeared within 4 months. The temporal and spatial trends of vegetative shoots densities were consistent with those of the total shoots (Figure 6B). Flowering shoots were observed from May to August at 1 m, 2 m, 3 m, and 4 m, and were also sporadically observed at 6 m (Figures 6C,E).

Shoot height at different depths increased gradually from May to August 2014 (Figure 6D). The maximum shoot height at 1 m and 2 m was ∼30 cm and ∼40 cm in September, respectively, and that at 3 m was ∼35 cm in August. Plant height began to decrease after summer, and it was ∼20 cm at the above-mentioned depths in November. However, the maximum shoot height at 4 m was ∼20 cm in September, and ∼10 cm in November (Figure 6D).

**FIGURE 7:**
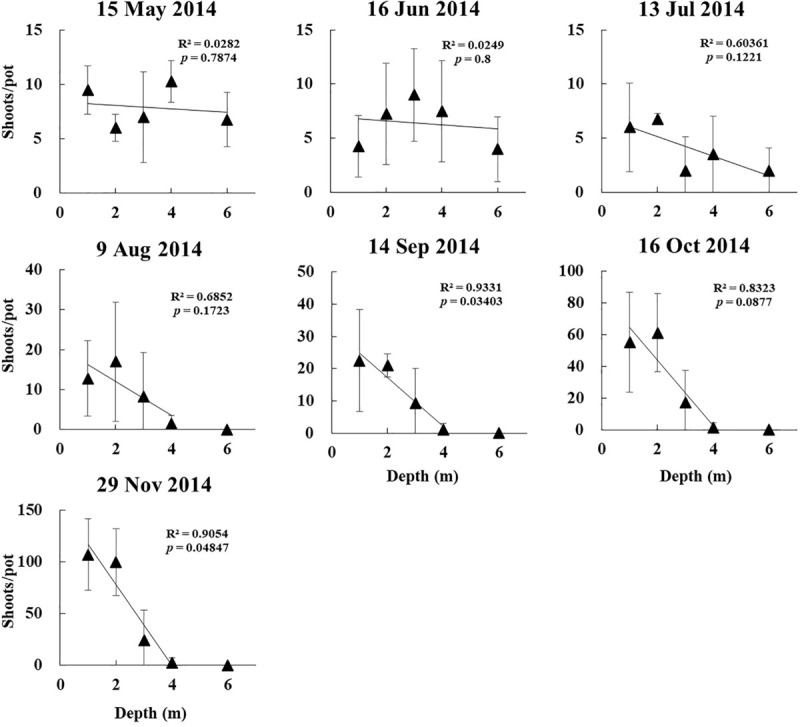
Linear regression of *Z. marina* vegetative shoot density vs. depth in a shoot transplantation experiments beginning in April 2014.

The linear regression of shoot density vs. depth revealed significant differences between depths in September and November for the 2014 experiment (*p* < 0.05, Figure 7).

### Correlations Between Water Parameters and Depth

**FIGURE 8:**
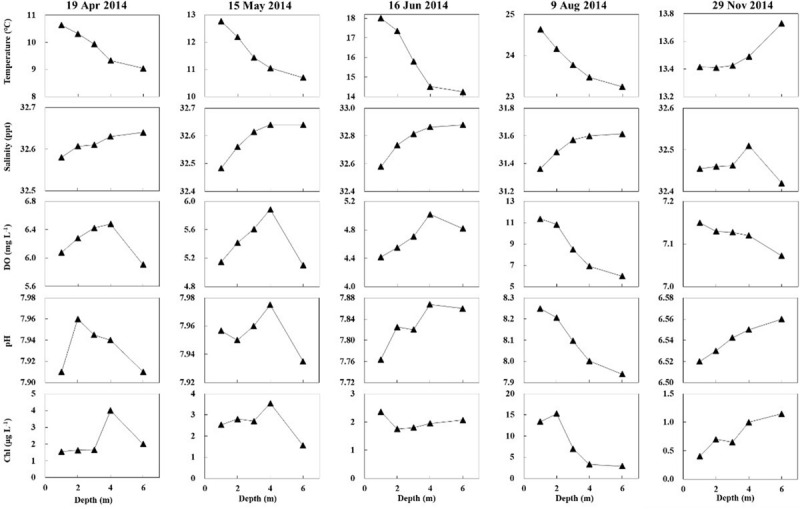
Changes in water temperature, salinity, dissolved oxygen (DO), pH, and chlorophyll content with depth in Ailian Bay in 2014.

**FIGURE 9:**
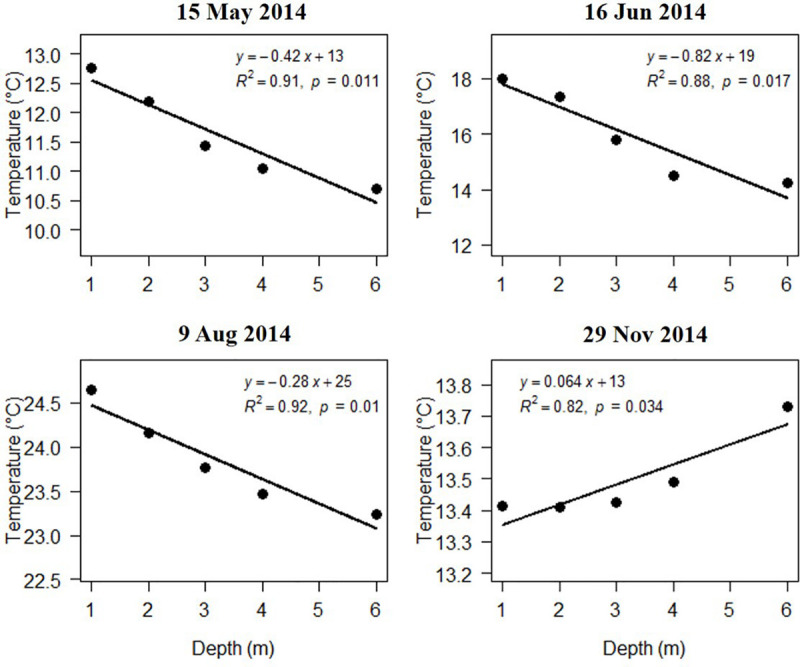
Variation in temperature with water depth in Ailian Bay at various dates during 2014.

**FIGURE 10:**
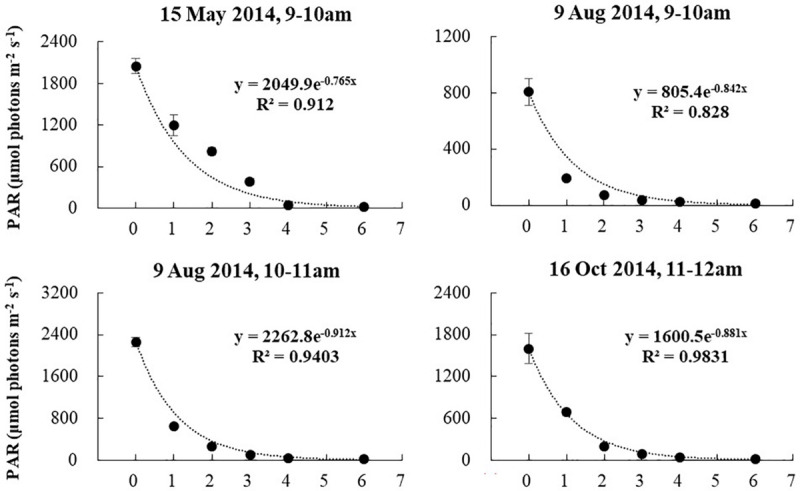
Variation in light availability with water depth in Ailian Bay at various dates and times during 2014. The fitted line is an exponent curve representing the fit of Beer’s law (equation 2) to the data.

Salinity, DO, and pH varied slightly with water depth. The chlorophyll content (Chl) changes with water depth were mostly slight. Temperature changes with water depth were relatively high in the warm season (April, May, June, and August 2014), ranging from 0.28 to 0.75°C/m (Figure 8). The regression of temperature vs. depth in May, June, August, and November were all significant (Figure 9; *p* < 0.05). Light intensity changed significantly as depth increased, and all four of the light vs. depth distributions adhered well to Beer’s Law (*R*^2^ ≥ 0.82; Figure 10). These results suggest that the water clarity in Ailian Bay did not substantially vary spatially with depth.

## Discussion

*Zostera marina* is a major habitat-forming species in north China. Its distribution may be threatened by changes in water clarity through increased sedimentation and nutrient input from agricultural and urban development (Thrush et al., 2004; Orth et al., 2006; Ralph et al., 2007). The present study defined the maximum depth limit for eelgrass (*Z. marina*) and provided a suitable depth range for successful restoration in local coastal waters, north China. More broadly, this study assessed the effect of light availability on the growth and recruitment of *Z. marina* conducted by *in situ* transplanted suspended cultures.

### *In situ* Suspended Cultures and Improvement of the Experimental Device

Site selection for potential seagrass transplantation is crucial for restoration success, and environmental parameters limit suitable habitat availability for seagrass restoration (van Katwijk et al., 2016). One essential parameter determining seagrass restoration success is depth (Aoki et al., 2020). However, suggestions for site selection for seagrass restoration are mainly based on either *in situ* investigations or laboratory experiments. Laboratory experimental systems remove important contextual factors under natural conditions (e.g., waves, episodic turbidity, and epiphyte growth) (Abal et al., 1994; Crain et al., 2008), and the minimum light requirement for eelgrass in laboratory experiments has been shown to be lower than that in the field (Short, 1991). For field investigations, results might be difficult to generalize for other locations because seagrass responses to light reduction are dependent on site-specific conditions (Lee et al., 2007).

To overcome inherent weaknesses of the abovementioned experiments, an *in situ* suspended culture experiment was developed in this study to directly examine long-term responses of transplanted *Z. marina* shoots to a depth gradient, in order to determine the depth limit for eelgrass restoration. Moreover, *in situ* suspended cultures may prove to be a useful method for seagrass research, e.g., studying the effect of habitat type on eelgrass seed germination (unpublished data). Over the 4 years of *in situ* suspended experiments, the stability of the experimental device was improved by switching from PE boxes to PVC pots, based on device drag force comparisons. The drag force of the PE box was approximately double to six-fold that of the PVC pot; indicating a greater stability of the PVC pot, and consequently none of these were overturned by marine current. In addition, because it is much easier for the PE box to rotate around its vertical axis under the influence of current, the PVC pot appears to be a more stable device for suspended cultures of eelgrass. For marine areas with strong currents, a heavier plumb ball can be added at the bottom of the experimental device to improve the stability of the system.

### Environmental Factors Contributing to the Depth Limit of *Z. marina*

Eelgrass shoot transplantation experiments provide evidence that eelgrass may have the ability to recolonize at an optimal depth of ≤3 m in Ailian Bay, where eelgrass has declined dramatically. The value of the *Z. marina* depth limit determined for Ailian Bay in this study was higher than that of the distribution depth (1–2 m; Xu et al., 2018, 2019) of natural populations at the donor site (Swan Lake). The depth limit in Ailian Bay is within the range reported previously, and *Z. marina* grows from the intertidal zone to depths of ∼10 m depending on water clarity (Jackson et al., 2013). However, even with sufficient light availability, seagrass can still decline if impacted by other environmental factors, such as increasing temperature (Marbà and Duarte, 2010) and high hydrogen sulfide content in sediment (Fraser and Kendrick, 2017). In China, land reclamation, clam harvesting, and mariculture have been suggested to be the main factors causing eelgrass habitat loss (Huang et al., 2006; Zheng et al., 2013).

Light availability, temperature, and inorganic nutrients are considered as major factors controlling seagrass growth (Lee et al., 2007). In the present study, light availability was identified as the most important factor determining the depth limit and growth of *Z. marina*, since there was little spatial variation vertically in other measured water parameters. In the present study, although *Z. marina* at 4 m depth persisted during the experimentation period, the shoot number, which was significantly lower than at shallow depths (≤3 m), appeared to show a downtrend. *Z. marina* at 4 m depth might disappear over a longer time scale with 1.75–2.88% of surface irradiance, since these values are lower than the minimum light requirement of 4–36% of surface irradiance for seagrass reported previously (Ralph et al., 2007). With 4.09–18.75% of surface irradiance, within the minimum light requirement of 4–36% (Ralph et al., 2007), the shoot number at 3 m depth presents seasonal variation with asexual reproduction in autumn. Therefore, *Z. marina* may be able to survival and recolonize at an optimal depth of ≤3 m in Ailian Bay over a longer time scale.

Sustained elevated temperatures can cause plant mortality (Hammer et al., 2018). Several studies have reported negative temperature effects on eelgrass morphology and survival over a range of 25–30°C (Orth and Moore, 1986; Touchette et al., 2003; Moore and Jarvis, 2008; Moore et al., 2014). The highest surface water temperature in Ailian Bay from August 2010 to September 2011 was 22.4°C, recorded by Liu (2012), indicating that eelgrass are not generally exposed to stressful temperatures in this area. Moreover, temperature has a small effect on eelgrass lateral branching (Eriander, 2017). For salinity, pH, and chlorophyll, the vertical variations in Ailian Bay were minimal, and thus likely have limited effects on seagrass productivity variation with depth (Lirman and Cropper, 2003). The DO concentrations at all depths were relatively high, except in June 2015 when phytoplankton abundance in the water column was high, leading to deeper areas experiencing anoxia (1–3 mg L^–1^) (Moore and Jarvis, 2008). Regarding nutrient concentrations, there are no significant differences between the surface water and bottom water (Li et al., 2018).

### Correlating *Z. marina* Depth Limit to Minimum Light Requirements

As shown in Figure 9, all four of the light vs. depth distributions adhered well to Beer’s Law (*R*^2^ ≥ 0.82), indicating that the variation in turbidity with water depth was not substantial. Since long-term measurements of subsurface photosynthetically active radiation were not recorded for the duration of the experiment, it is difficult to convert the estimated depth threshold of 3 m to a similarly precise light threshold for *Z. marina*. However, combining all estimated values of *K_*d*_^∗^* shown in Figure 9, the light experienced at 3 m depth was between 6.48 and 10.08% of the surface irradiance. These values agree well with the minimum light requirement of 4–36% of surface irradiance for seagrass reported previously (Ralph et al., 2007).

### Shoot Density as an Indicator of Seagrass Response to Light Reduction

*In situ* reductions of light are known to affect seagrasses in a variety of ways. Several studies have shown stunted growth, reduced biomass, and lower densities in response to reduced light (Abal et al., 1994; Collier et al., 2009, 2016). Shoot density is determined by lateral shoot production (Olesen and Sand-Jensen, 1994a), thus branching frequency is also reduced at low light availability. The production of new lateral branching shoots is essential for restoration success, and a high lateral branching frequency would aid in the establishment and expansion of transplants, thereby reducing the risk of failure due to stochastic events (Olesen and Sand-Jensen, 1994b). Furthermore, the present experiment conducted in autumn 2011 demonstrated shoot loss of 60–82.5% at ≤4 m depth over the winter of 2011, increasing the risk of complete transplant mortality over winter, and indicating that high lateral shoot production in autumn of 2012 may be essential for reducing the risk. Therefore, it would be more useful, for restoration purposes, to determine the minimum light requirement for high lateral branching rather than for general growth and maintenance (Eriander, 2017).

### Implications for Restoration

Depth is a critical determinant of seagrass restoration success (Aoki et al., 2020). These results suggest that the greatest eelgrass survival may occur in areas ≤3 m depth, which are therefore the most suitable sites for restoration in Ailian Bay. Moreover, the current transplantation experiments have demonstrated greater survival and lateral branching frequency of shoots when restoration occurs in the spring (2014) than in the autumn (2011). Considering the similar conclusions reported by previous researchers, this indicates that restoration trials occurring in the spring and early summer will result in the greatest survival of shoots (Vichkovitten et al., 2007). This is because transplants have sufficient time to acclimatize, store carbohydrates, grow, and undergo lateral shoot branching during the first season (Eriander, 2017). In addition, the present findings suggest that the technique of suspending cultures of eelgrass, can be used to improve water quality, through regulating nutrient cycles, and attracting fish through the provision of food (e.g., zooplankton) and habitats.

## Conclusion

This study presented a novel method (*in situ* suspended cultures) to directly examine long-term responses of transplanted *Z. marina* shoots to a depth gradient, in order to determine the depth limit (light requirement) for eelgrass restoration. The findings indicate that adult *Z. marina* transplants originating from a nearby matching donor site (Swan Lake) can successfully acclimatize and be used for restoration within degraded areas (Ailian Bay) requiring eelgrass restoration. These results provide a suitable depth range for successful restoration of eelgrass in Ailian Bay, north China. More broadly, this work may provide useful knowledge for the global management and restoration of seagrass.

## Data Availability Statement

The raw data supporting the conclusions of this article will be made available by the authors, without undue reservation.

## Author Contributions

SX: writing – original draft, conceptualization, methodology, investigation, and validation. PW, FW, PL, and BL: investigation, formal analysis, and writing – review and editing. XZ: data curation, formal analysis, investigation, and writing – review and editing. SY and YuZ: investigation and data curation. YiZ: funding acquisition, supervision, methodology, investigation, and writing – review and editing. All authors contributed to the article and approved the submitted version.

## Conflict of Interest

The authors declare that the research was conducted in the absence of any commercial or financial relationships that could be construed as a potential conflict of interest.
